# Incomplete Antibodies May Reduce ABO Cross-Match Incompatibility: A Pilot Study

**DOI:** 10.4274/tjh.2016.0504

**Published:** 2018-03-06

**Authors:** Mehmet Özen, Soner Yılmaz, Tülin Özkan, Yeşim Özer, Aliye Aysel Pekel, Asuman Sunguroğlu, Günhan Gürman, Önder Arslan

**Affiliations:** 1 Ufuk University Faculty of Medicine, Department of Hematology, Ankara, Turkey; 2 University of Health Sciences, Gülhane Training and Research Hospital, Blood Bank Unit, Ankara, Turkey; 3 Ankara University Faculty of Medicine, Department of Medical Biology, Ankara, Turkey; 4 Ankara University Faculty of Medicine, Unit of Blood Bank, Ankara, Turkey; 5 University of Health Sciences, Gülhane Training and Research Hospital, Clinic of Immunology and Allergy Diseases, Ankara, Turkey; 6 Ankara University Faculty of Medicine, Department of Hematology, Ankara, Turkey

**Keywords:** Transfusion medicine, Red blood cells, Complications, Humoral immune response

## Abstract

**Objective:**

Any erythrocyte transfusion among humans having type A or B blood groups is impossible due to antibodies causing fatal transfusion complications. A cross-match test is performed to prevent immune transfusion complications before transfusion. Our hypothesis is that the fragment antibody (Fab) part of the antibody (incomplete antibody) may be used to prevent an immune stimulus related to the complete antibody. Therefore, we designed a pilot study to evaluate the effectiveness of these incomplete antibodies using cross-match tests.

**Materials and Methods:**

Pepsin enzyme and staphylococcal protein A columns were used to cut anti-A and anti-B monoclonal antibodies and purify their Fab (2) fragments, respectively. An Rh-positive erythrocyte suspension with purified anti-A Fab (2) solution and B Rh-positive erythrocyte suspension with purified anti-B Fab (2) solution were combined correspondingly. Cross-match tests were performed by tube and gel centrifugation methods. The agglutination levels due to the anti-A and anti-B Fab (2) antibodies and their effects on the agglutination normally observed with complete antibodies were then measured.

**Results:**

No agglutination for the purified incomplete anti-A Fab (2) with A Rh+ erythrocyte and anti-B Fab (2) with B Rh+ erythrocyte combinations was observed in the tube cross-match tests. These agglutination levels were 1+ in two wells in the gel centrifugation cross-match tests. Fab (2)-treated erythrocytes were also resistant to the agglutination that normally occurs with complete antibodies.

**Conclusion:**

We determined that the Fab (2) fragments of antibodies may not only be used to obtain a mild or negative reaction when compared to complete antibodies, but they might also be used for decreasing ABO incompatibility. Incomplete antibodies might be a therapeutic option in autoimmune hemolytic anemia and they may also be used in solid organ or hematopoietic stem cell transplantation. Therefore, we have planned an in vivo study to prove these in vitro findings.

## Abstract (tr)

**Amaç:**

Tip A ve B kan grubuna sahip insanlar arasında herhangi bir eritrosit nakli öldürücü transfüzyon komplikasyonlarına neden olan antikorlar nedeniyle imkansızdır. İmmün transfüzyon komplikasyonlarını önlemek için transfüzyondan önce çapraz karşılaştırma testi yapılır. Hipotezimiz komplet antikorla ilişkili bağışıklık yanıtını önlemekte antikorun fragman antikor (Fab) parçasının (inkomplet antikor) kullanılabileceğidir. Bu inkomplet antikorların etkinliğini değerlendirmek için de çapraz karşılaştırma testlerini kullanarak bir başlangıç çalışması tasarladık.

**Gereç ve Yöntemler:**

Anti-A ve anti-B monoklonal antikorlarını kesmek ve saflaştırmak için sırasıyla pepsin enzimi ve stafilokokal protein A kolonları kullanıldı. A Rh pozitif eritrosit süspansiyonu ile saflaştırılmış anti-A Fab (2) solüsyonu ve B Rh pozitif eritrosit süspansiyonu ile saflaştırılmış anti-B Fab (2) solüsyonu sırasıyla birleştirildi. Çapraz karşılaştırma testleri tüp ve jel santrifügasyon yöntemleri kullanılarak çalışıldı. Sonrasında anti-A ve anti-B Fab (2) antikorlara bağlı aglütinasyon düzeyi ve bunların komplet antikorlarla normalde gözlenen aglütinasyon üzerine etkileri ölçüldü.

**Bulgular:**

Tüp yöntemi ile yapılan çapraz karşılaştırma testinde saflaştırılmış inkomplet anti-A Fab (2) ile A Rh pozitif eritrosit ve anti-B Fab (2) ile B Rh pozitif eritrosit kombinasyonlarında aglütinasyon gözlenmedi. Jel santrifügasyon yöntemi ile yapılan çarpraz karşılaştırma testinde bu aglütinasyon düzeyleri her iki kuyucukta da 1 pozitifti. Fab (2) ile muamele edilen eritrositler komplet antikorla normalde oluşan aglütinasyona da dirençliydiler.

**Sonuç:**

Antikorların Fab (2) fragmanlarının sadece komplet antikorlara kıyasla daha hafif veya negatif reaksiyonu elde etmekte değil, aynı zamanda ABO uyumsuzluğunu azaltmakta da kullanılabileceğini değerlendirdik. İnkomplet antikorlar otoimmün hemolitik anemide bir tedavi seçeneği olabileceği gibi aynı zamanda solid organ veya hematopoeitik kök hücre naklinde kullanılabilir. Bu nedenle in vitro bulguları doğrulamak için in vivo bir çalışma planladık.

## Introduction

There are many blood groups used for the human population, including ABO, Rh, Kidd, Kell, Duffy, MNS, and Lewis. The ABO system is the most important of all blood groups in transfusion practice due to the reciprocal antibodies [1]. These antibodies consistently and predictably present in the sera of normal people whose erythrocytes lack the corresponding antigen(s) [2]. These antibodies may cause immediate lysis of donor red blood cells (RBCs) during ABO-incompatible transfusion and initiate fatal hemolytic transfusion reactions [1].

Typing and screening are the first steps of pretransfusion compatibility tests. These tests are used to define the patient’s ABO group and Rh type and to detect expected and unexpected antibodies in the patient’s serum. The cross-match is the final step of pretransfusion testing [3]. In this test, donor cells are combined with the patient’s serum and checked for agglutination, which would signify incompatible blood [4]. This process, also known as major cross-matching, serves as the last safeguard to ensure a safe transfusion [4,5,6].

Antibodies are also essential for humoral immunity [7]. Many antibodies have been shown to be primarily related to autoimmune diseases and such diseases are referred to as antibody-related autoimmune diseases [7,8,9]. Many of these diseases may disappear in the absence of certain antibodies [9]. 

All antibodies have two fragments. The antigen-binding fragment (Fab) binds to an antigen, and the crystallizable fragment (Fc) stimulates the immune system by activating the complement [10]. Additionally, macrophages or lymphocytes detect the Fc fragment of antibodies [11,12]. Therefore, the Fab fragment detects antigens and the Fc fragment stimulates the immune system. An antibody with the Fc part removed, in which only the Fab fragment exists, may be called an incomplete antibody. Papain or pepsin enzymes can be used in the fragmentation of antibodies and can produce Fab or Fab (2) fragments of the antibodies, respectively [13]. The effectiveness levels of Fab and Fab (2) fragments of an antibody are similar and they are interchangeable [14]. Our hypothesis is that incomplete antibodies may be used to prevent an immune stimulus. We designed a pilot study to examine the effectiveness of these incomplete antibodies in incompatible cross-matches due to ABO antibodies and we are presenting it here. Local ethics committee approval was obtained for this study.

## Materials and Methods

Anti-A and anti-B monoclonal antibodies (Eryclone, Verna Industrial Estate, Verna, India) were used for this study. First the pepsin enzyme was used to cut these monoclonal antibodies and staphylococcal protein A columns were used to purify their Fab (2) fragments. The Pierce™ F(ab’)2 Preparation Kit (Thermo Fisher Scientific, Rockford, IL, USA) was used to produce the Fab (2) fragments from complete antibodies. This process was conducted according to the manufacturer’s instructions. 

After obtaining purified Fab (2)s, we began the second part of the study. During purification of Fab (2)s, the volume of the products changed. The ratios of the complete monoclonal antibodies to the standard erythrocyte solution for an optimal cross-match test were calculated according to the manufacturer’s instructions. We used these ratios for the anti-A or -B Fab (2) to the A or B Rh-positive erythrocyte solutions for the cross-match tests, respectively. 

After calculation, an anti-IgG cross-match card (Ortho-Clinical Diagnostics, High Wycombe, UK) was used for the compatibility tests. We combined 10 µL of A Rh-positive 5% erythrocyte suspension with 150 µL of purified anti-A Fab (2) solution in the same well to conduct a cross-match test in order to prove that the erythrocytes were covered with anti-A Fab (2). We also used 150 µL of complete anti-A antibodies for the positive control and 150 µL of phosphate-buffered saline (PBS) for the negative control. We incubated all cards at 37 °C for 10 min and then centrifuged them for 5 min. The negative controls lacked the complete and incomplete antibodies. We repeated the same process with complete and incomplete anti-B antibodies and the B Rh-positive erythrocyte suspension. In addition, we repeated these tests using Across Gel^®^ Anti-Human Globulin IgG+C3d cross-match cards (Dia Pro, İstanbul, Turkey). We also evaluated agglutination levels when complete and incomplete antibodies were put in the same well at the same time, noting the amounts for A and B erythrocyte suspensions. We conducted an antibody titration test and repeated this last test with several ratios (32/1, 8/1, 4/1, 1/1, 1/4, and 1/16) for complete to incomplete antibodies when used simultaneously. Finally, we evaluated the reactions in all wells. 

We also performed a tube test to confirm the results of the card tests and to show whether incomplete antibodies inhibited normal agglutination with complete antibodies or not. First, we treated A Rh+ erythrocytes with anti-A Fab (2) and B Rh+ erythrocytes with anti-B Fab (2) in two separate tubes. We then added complete anti-A and anti-B antibodies to the respective tubes and mixed them. As a positive control, A Rh+ erythrocytes were treated only with complete anti-A antibodies and B Rh+ erythrocytes were treated only with complete anti-B antibodies in a tube without adding incomplete fragments. Consequently, there were no incomplete antibodies in positive control tubes. We then evaluated the agglutination levels in the tubes. 

In addition, we performed a flow cytometric analysis to prove the results of all these tests. The B Rh+ erythrocyte sample was transferred to a tube containing K_3_ EDTA and that tube’s contents were divided into four tubes. We mixed each tube with one of the following: PBS, anti-B complete antibodies alone, anti-B incomplete antibodies alone, or a mix of anti-B antibodies (1:1 ratio for incomplete to complete). To label the erythrocytes, CD235a FITC (glycophorin A, BD Pharmingen, San Diego, CA, USA) and cytoplasm-staining nucleic acid dye 7-amino-actinomycin (7-ADD) (BD Pharmingen) were added to the tubes. The samples were analyzed using the FACSDiva software of the FACSCanto II model flow cytometer (BD Biosciences, San Jose, CA, USA). Viable erythrocytes were identified as cells stained positive with CD235a FITC and negative with 7-ADD. We evaluated 100,000 events per sample to show the erythrocyte agglutination levels in the tubes. Agglutination levels were calculated with the single-cell analysis and forward-scatter gating strategy [15].

## Results

**Figure 1:**
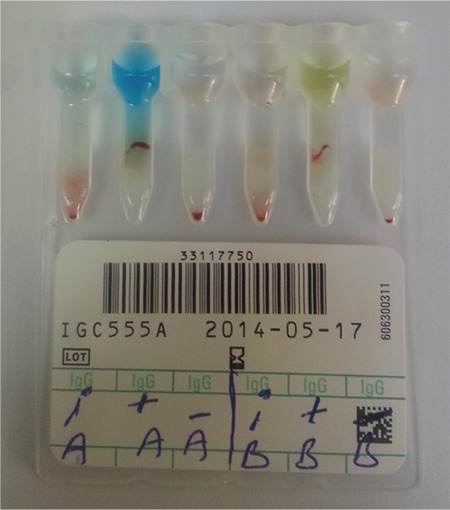
A) Group A erythrocytes: Ai, with incomplete anti-A antibody (1+ reaction); A+, with complete anti-A antibody (4+ reaction); A-, with negative control (no reaction). B) Group B erythrocytes: Bi, with incomplete anti-B antibody (1+ reaction); B+, with complete anti-B antibody (4+ reaction); B-, with negative control (no reaction).

**Figure 2:**
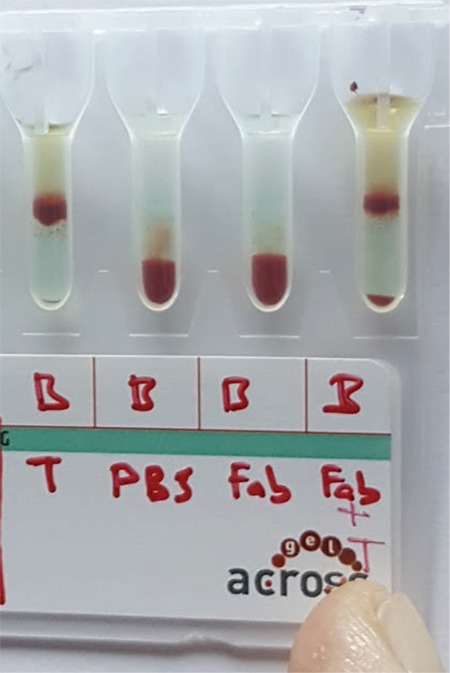
Group B erythrocytes: T, with complete (total) antibody (4+ reaction); PBS, with phosphate-buffered saline (- reaction); Fab, with anti-B Fab (2) (- reaction); Fab+T: with anti-B complete (total) and anti-B Fab (2) simultaneously, 1:1 dilution (double reaction with 4+ and -). *PBS: Phosphate-buffered saline, Fab: fragment antibody.*

For the card test, we observed a 1+ reaction for the purified incomplete anti-A Fab (2) and A Rh+ erythrocyte combination. However, we observed 4+ reactions for the complete anti-A antibody with the A Rh+ erythrocyte combination. No positive reactions were observed in the negative control wells. The test results were similar for the B Rh+ erythrocyte and complete anti-B or incomplete anti-B Fab (2) antibody combinations and negative controls (Figures 1 and 2). 

The antibody titration test results are given in Table 1. Higher concentrations of complete antibodies (from 8 to 32 times more than incomplete antibodies) were associated with 4+ agglutination levels in simultaneous use on the cross-match card tests (Table 1). Lower ratios than 8/1 showed double population results when both complete and incomplete antibodies were simultaneously added to the wells before erythrocytes (Table 1, Figure 2). Increasing the amounts of incomplete antibodies did not cause any 4+ results if complete antibodies were not added to the wells. 

**Figure 3:**
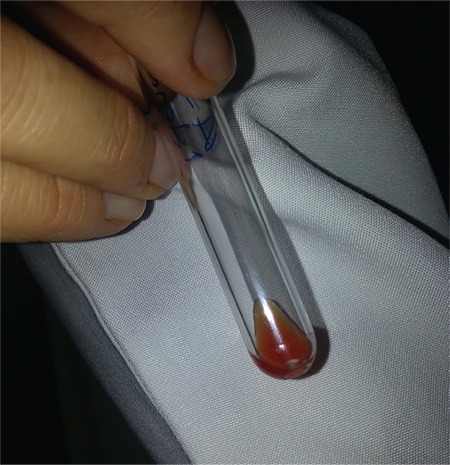
Group A and group B erythrocytes in the same tube incubated with incomplete anti-A and -B fragment antibody fragments and after addition of complete anti-A and -B to the medium. No agglutination.

**Figure 4:**
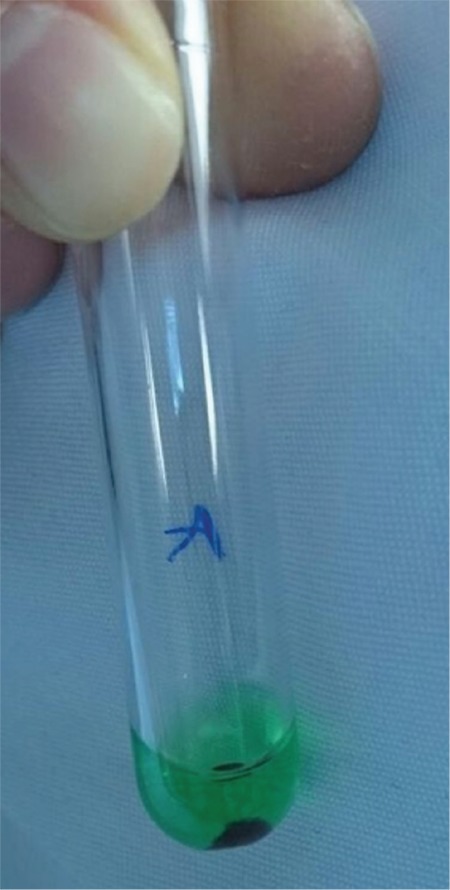
Group A and group B erythrocytes in the same tube incubated with complete anti-A and -B antibodies. Positive agglutination.

For the tube tests, we observed no agglutination for the A Rh+ erythrocytes and incomplete anti-A Fab (2) antibodies combination and the B Rh+ erythrocytes and incomplete anti-B Fab (2) antibodies combination in two separate tubes. There was also no agglutination when complete anti-A and anti-B antibodies were added to the respective tubes. No agglutination continued when the two tubes were mixed (Figure 3). Agglutination was present in the positive control tube that contained complete antibodies (Figure 4). 

**Figure 5:**
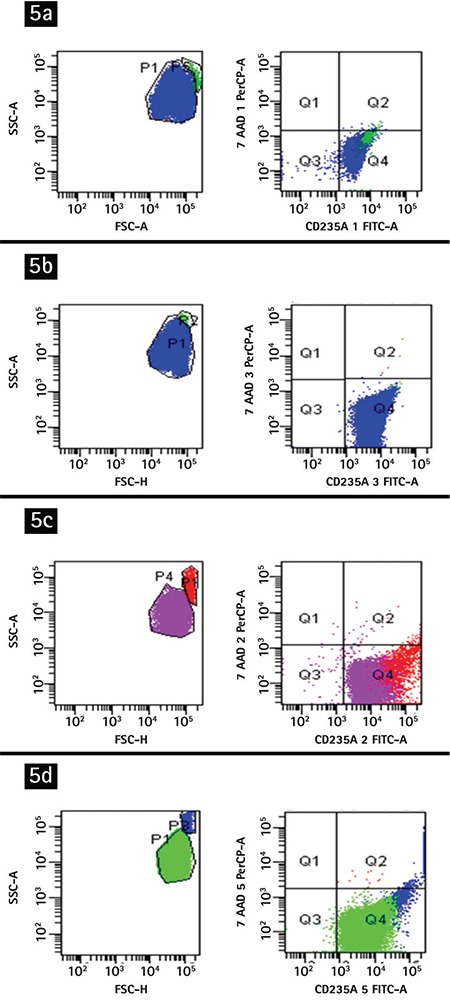
Flow cytometric analysis with group B erythrocytes: a) with phosphate-buffered saline, 0.9% agglutination; b) with anti-B fragment antibody (Fab) (2), 0.1% agglutination; c) with complete anti-B, 7.1% agglutination; d) with anti-B complete and Fab (2) simultaneously, 2.9% agglutination.

Flow cytometric analysis also showed similar results (Figure 5). Agglutinated erythrocytes expressed brighter CD235a positivity than non-agglutinated erythrocytes. Almost all erythrocytes were viable in the tubes. Erythrocyte agglutination levels were calculated as 0.9% for the PBS tube, 0.1% for the Fab (2) tube, 7.1% for the complete anti-B antibody tube, and 2.9% for the mixed tube (1:1, complete to incomplete antibody).

## Discussion

Although complete anti-A and -B antibodies cause strong agglutination, the Fab (2) parts of these antibodies caused minimal or no agglutination in the card and tube cross-match tests. Minimal agglutination with Fab (2) parts was similar to the negative controls. These results come from the characteristic features of an antibody. The Fab part of an antibody binds to the antigen, and the Fc part of the antibody both starts agglutination and stimulates the immune system via activating the complement system and/or binding to Fc receptors of macrophages or lymphocytes [10,11,12]. Fc and its interactions with the Fc receptors of macrophages have a critical role and are required for antibody response [16,17]. Hemolytic disease of newborns is a good example of this pathologic mechanism of antibody response. In this antibody-related disease, anti-D antibody treatment is used to prevent hemolytic disease of newborns [18]. Anti-D antibody drugs should be composed of complete antibodies to prevent competitive binding of Fab fragments [16]. In the past, purified Fab fragments of the anti-D antibody were studied for hemolytic disease of newborns because of their binding to Rh+ erythrocytes [19,20]. However, anti-D Fab treatment was not sufficient for being used for hemolytic disease of newborns due to its ineffectiveness [16]. This situation comes from the Fc part of the antibody. Removal of the Fc part of an antibody may result in ineffectiveness of the antibody when stimulating the immune system even if it binds to an antigen. Similarly, the digoxin-specific incomplete Fab antibody effectively binds to its antigen (Digifab). However, no significant immune reaction was reported in patients treated with this agent, probably due to the absence of the Fc part of the antibody [21].

ABO incompatibility is an unavoidable clinical issue, and complications associated with ABO incompatibility should be managed and treated appropriately [22]. Hemovigilance procedures are recommended and used because of the potential for fatal complications following blood transfusion [23]. Although some procedures for treating ABO-incompatible blood transfusions are used, to the best of our knowledge, none of them are specific [24]. In our study, we showed that if erythrocytes are exposed to Fab (2) and complete antibodies simultaneously, complete antibody-associated agglutination ratios may decrease due to the coating of some erythrocytes with Fab (2) and others with complete antibodies. Therefore, we hypothesized that anti-A and anti-B Fab (2) antibodies might be a useful treatment for these patients and may reduce fatal complications. Competitive binding between complete and incomplete antibodies may reduce or eliminate the effects of complete antibodies [25]. No strong agglutination with high amounts of Fab (2) and insufficiency of low amounts of Fab (2) in preventing agglutination related to complete antibodies also supports our hypothesis. Similarly, anti-Rh antibodies are considered for use in preventing transfusion reactions in hemolytic disease of newborns and their effect is superior when they are used early after birth [26]. Our results could also be explained with the epitope-masking hypothesis [27]. When an epitope on an antigen is coated with an antibody, other antibodies cannot bind the same epitope. Therefore, if the first antibody did not start an immune response and occupy the epitope, the following antibodies will also not be able to cause an immune response. Our hypothesis may be stated as follows: the Fab (2) parts of the same antibodies may be used for masking the epitopes instead of other antibodies. Moreover, our findings may also help in universal group O RBC studies [28]. Instead of polyethylene glycol, Fab (2)s may be used in order to cover erythrocytes via coating surface antigens. However, our in vitro study needs to be supported with future in vivo animal studies for this use. Therefore, we are planning to conduct an in vivo study to prove the results of our pilot study.

ABO incompatibility between the donor and the recipient can cause hemolysis in the recipient, especially when performing hematopoietic and solid organ transplantations [22,29]. It also presents several challenges for hematopoietic stem cell transplantation [29]. During hematopoietic stem cell transplantation, transfused erythrocytes and other blood products change based on the donor’s and recipient’s blood groups, and such changes are not stable [30]. Irradiated, filtered, and leukocyte-depleted blood products are commonly used for blood transfusions [31]. Some hemolytic anemia patients also have auto-anti-A or auto-anti-B antibodies [32,33,34]. We hypothesize that the anti-A and anti-B Fab (2) antibody fragments presented here may be used to prepare suitable or alternative blood products for such patients in the future. Using Fab (2) fragments of antibodies, including those of other blood groups, may simplify current antibody screening and identification tests. In spite of the importance of these tests, due to problems originating from technical procedures and evaluation methods, these tests take time, postpone the use of blood products for patients, and sometimes result in inconclusive outcomes [35]. As we showed, seeing a double population in a well may help in the identification process of antibodies.

ABO-incompatible solid organ transplantation presents other challenges, and some such transplants are currently impossible due to ABO incompatibility [22,36]. Solid organs contain ABO antigens that can cause incompatibility [22,36]. Immunoadsorption techniques are used to prevent the antibody-related immune response and to extend the survival of grafts and transplant recipients having ABO incompatibility [37]. Hyperacute rejection in solid organ transplants may also be reversed by using Fab fragments [38]. We hypothesized that the intravenous administration of anti-A and anti-B Fab (2) antibody fragments may also be applied in solid organ transplantation.

### Study Limitations

Our study has some limitations. Our sole aim was to test our hypothesis that Fab (2) antibody fragments can be used to prevent an immune stimulus. All of the funds for this project were provided by the authors and our funds were not sufficient to fully complete the project. Although the results of cross-match tests and flow cytometric analysis were consistent, we were not able to evaluate all possible immune stimulus mechanisms associated with incomplete antibodies. In addition, we would have preferred to measure the levels of the Fab (2) antibody fragments and pepsin after completing the reaction, and also to measure the reaction in various environmental conditions, but we did not have sufficient funds to perform all these tests. It should also be noted that the weak positive reactions in group A or B erythrocytes with incomplete anti-A or anti-B antibodies in IgG cross-match card tests, respectively, may have originated from inadequate Fab (2) antibody fragment yields with pepsin and protein A columns in our study [39]. However, we cannot state a definite reason explaining these mild agglutinations in card tests as we could not measure the levels of complete and incomplete antibodies in the products used for card tests. Higher agglutination results related to Fab (2) fragments by the gel centrifugation technique than tube tests may also have originated from its higher sensitivity in detecting agglutination [40].

## Conclusion

In this in vitro study, we showed that ABO incompatibility can be minimized by using Fab (2) antibody fragments of anti-A and anti-B antibodies. In vivo studies are needed to explore the potential therapeutic effects of these agents. Therefore, we have planned to start an in vivo study to prove these in vitro findings.

## Figures and Tables

**Table 1:**
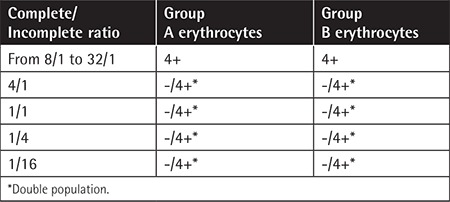
Antibody titration tests on the immunoglobulin G cross-match cards according to the ratios for complete to incomplete antibodies added simultaneously.
